# Environmental Nutrient Supply Directly Alters Plant Traits but Indirectly Determines Virus Growth Rate

**DOI:** 10.3389/fmicb.2017.02116

**Published:** 2017-11-06

**Authors:** Christelle Lacroix, Eric W. Seabloom, Elizabeth T. Borer

**Affiliations:** Department of Ecology, Evolution, and Behavior, University of Minnesota, Saint Paul, MN, United States

**Keywords:** nutrient supply, stoichiometry, coinfection, plant traits, virus accumulation, transmission

## Abstract

Ecological stoichiometry and resource competition theory both predict that nutrient rates and ratios can alter infectious disease dynamics. Pathogens such as viruses hijack nutrient rich host metabolites to complete multiple steps of their epidemiological cycle. As the synthesis of these molecules requires nitrogen (N) and phosphorus (P), environmental supply rates, and ratios of N and P to hosts can directly limit disease dynamics. Environmental nutrient supplies also may alter virus epidemiology indirectly by changing host phenotype or the dynamics of coinfecting pathogens. We tested whether host nutrient supplies and coinfection control pathogen growth within hosts and transmission to new hosts, either directly or through modifications of plant tissue chemistry (i.e., content and stoichiometric ratios of nutrients), host phenotypic traits, or among-pathogen interactions. We examined two widespread plant viruses (BYDV-PAV and CYDV-RPV) in cultivated oats (*Avena sativa)* grown along a range of N and of P supply rates. N and P supply rates altered plant tissue chemistry and phenotypic traits; however, environmental nutrient supplies and plant tissue content and ratios of nutrients did not directly alter virus titer. Infection with CYDV-RPV altered plant traits and resulted in thicker plant leaves (i.e., higher leaf mass per area) and there was a positive correlation between CYDV-RPV titer and leaf mass per area. CYDV-RPV titer was reduced by the presence of a competitor, BYDV-PAV, and higher CYDV-RPV titer led to more severe chlorotic symptoms. In our experimental conditions, virus transmission was unaffected by nutrient supply rates, co-infection, plant stoichiometry, or plant traits, although nutrient supply rates have been shown to increase infection and coinfection rates. This work provides a robust test of the role of plant nutrient content and ratios in the dynamics of globally important pathogens and reveals a more complex relationship between within-host virus growth and alterations of plant traits. A deeper understanding of the differential effects of environmental nutrient supplies on virus epidemiology and ecology is particularly relevant given the rapid increase of nutrients flowing into Earth's ecosystems as a result of human activities.

## Introduction

Ecological stoichiometry (Sterner and Elser, 2002; Hessen et al., 2013) and resource competition (MacArthur, 1972; Tilman, 1982; Miller et al., 2005) are two powerful theoretical frameworks for understanding the effects of altered nutrient supplies on the physiology and ecology of organisms. Both frameworks rest on the observation that species differ in their requirements for the supply rates and ratios of different elemental nutrients. Ecological stoichiometry primarily is founded on the assumption that the stoichiometric balance, or ratio, of multiple chemical elements (e.g., carbon [C], nitrogen [N], and phosphorus [P]) available to organisms is a driver of ecological processes (Sterner and Elser, 2002; Hessen et al., 2013; Hillebrand et al., 2014). In particular, this framework has been used to study the effects of C:N:P ratios in a resource (e.g., prey) on consumers' growth (Sterner and Elser, 2002; Hessen et al., 2013; Hillebrand et al., 2014). In contrast, resource competition theory predicts population persistence, population growth, and species coexistence based on both rates and ratios of nutrient resources and is based on the assumption of competitive interactions between species (e.g., consumers and prey) for shared resources (MacArthur, 1972; Miller et al., 2005). While originally grounded in aquatic and marine ecosystems (Redfield, 1958; Corner et al., 1976; Tilman, 1976, 1977), both ecological stoichiometry and resource competition have been used to assess the effects of nutrient addition on the abundance, diversity, and functional traits of coexisting species of free-living organisms in terrestrial ecosystems (Haddad et al., 2000; Cardinale et al., 2009; Zehnder and Hunter, 2009; Elser et al., 2010; Borer et al., 2014a,b; Seabloom et al., 2015a).

In addition to free-living organisms, ecological stoichiometry (Sterner and Elser, 2002) and resource competition (Miller et al., 2005) also can be used to interpret the effect of nutrient supply rates and ratios on microbe dynamics (Smith, 1993, 2007; Smith and Holt, 1996; Aalto et al., 2015). Both theoretical frameworks predict that changes in host nutrient supplies can alter microorganismal reproduction (i.e., titer or population size), because host nutrient content can limit the production of nutrient-demanding microbial cells and particles (Smith, 2007). Consistent with these predictions, the growth rate of human and animal microorganisms, algae viruses, and bacteria in crustaceans has been shown to be linked to nutrient stoichiometry (Elser et al., 2003; Karpinets et al., 2006; Clasen and Elser, 2007; Frost et al., 2008; Lange et al., 2014; Maat and Brussaard, 2016). Nutrient supply rates and ratios also can control the dynamics of various plant and insect infectious diseases (Mitchell et al., 2003; Bedhomme et al., 2004; Borer et al., 2010, 2014b; Seabloom et al., 2013, 2015b; Lacroix et al., 2014). However, whether environmental resource supplies influence the ecology of plant viruses at different stages of the infection cycle and through direct effects on virus growth or indirect effects on partners (i.e., host plant and competitors) of the interactive network leading to epidemics remains unclear.

Applying stoichiometric and resource competition theory to host-microbe interactions may not be straightforward, because environmental nutrient supplies and ratios available to hosts may alter disease dynamics at different stages of the epidemiological cycle (i.e., infection success after inoculation, within host-multiplication, and between-host transmission; Aalto et al., 2015; Seabloom et al., 2015b; Borer et al., 2016). In addition, each of these stages can be influenced through a variety of pathways (Borer et al., 2016) including: (i) direct effects of nutrient addition to hosts on the rates and ratios of limiting nutrients available for within-host pathogen replication (Smith et al., 2005), (ii) indirect effects of nutrient availability to the focal pathogen mediated by interactions with other competing pathogens (Smith and Holt, 1996; Smith, 2007; Lacroix et al., 2014; Lange et al., 2014), and (iii) indirect effects of nutrient supply mediated by changes in host growth rates, size, and other functional traits (Whitaker et al., 2015).

Ecological stoichiometry (Sterner and Elser, 2002) and resource competition (Miller et al., 2005) theory predict that the content and ratios of nutrients available in hosts could directly limit the production of molecules such as nucleic acids and proteins that are necessary for the infection cycle of micro-organisms. Obligate parasites such as plant viruses rely entirely on their host to complete multiple steps of their epidemiological cycle, including host entry and within-host accumulation; and within hosts, viruses hijack host nitrogen- and phosphorus- rich molecules and metabolic pathways (Maule et al., 2002; Sterner and Elser, 2002; Ahlquist et al., 2003; Elser et al., 2010). In controlled conditions, environmental supply rates, but not ratios, of N and P to plant hosts increase the probability of successful infection establishment (Bawden and Kassanis, 1950a; Lacroix et al., 2014; Smith, 2014). Within-host accumulation rate also can be controlled by host resources and host nutrient stoichiometry (Spencer, 1941; Bawden and Kassanis, 1950b; Adam et al., 1987; Eraslan et al., 2007; Dordas, 2009; Alexander, 2010; Rua et al., 2013). Further, as within-host pathogen population growth often has been correlated with transmission rate to new hosts (Froissart et al., 2010), changes in within-host growth driven by host nutrient supplies could alter secondary transmission events and disease dynamics.

Host nutrient supply rates and ratios also could act indirectly on pathogen within-host growth and between-host transmission by influencing coexistence between coinfecting pathogens because of inter-specific variation in stoichiometric C:N:P requirements (Jover et al., 2014; Smith, 2014; Aalto et al., 2015) and also could alter interactions among the community of pathogens within a host. Plants may host many micro-organisms (Seabloom et al., 2009; Roossinck, 2012), and inter-specific microbial interactions within a host can range from antagonistic to neutral to facilitative with various potential consequences for disease dynamics (Turner, 2005; Rigaud et al., 2010; Elena et al., 2014; Seabloom et al., 2015b). For example, increased supplies of nitrogen can reduce among-virus competition and increase infection success and coinfection rates (Lacroix et al., 2014). Nutrient competition among microbes sharing a host also can change disease dynamics by altering within host accumulation, virulence (i.e., detrimental effects of infection on host fitness), transmission rates and disease emergence (Smith and Holt, 1996; Al-Naimi et al., 2005; Pedersen and Fenton, 2007; Alizon et al., 2013; Hall and Little, 2013; Salvaudon et al., 2013; Lange et al., 2014; Borer et al., 2016).

Environmental nutrient supplies also may alter pathogen dynamics indirectly at various stages of the infection cycle by changing host functional traits. For example, N has been shown to increase the concentration of a plant virus through its impacts on host biomass, rather than via direct effects on the virus (Whitaker et al., 2015). Functional traits corresponding to host morphological, physiological, and phenological properties can ultimately impact organisms' fitness in varying environmental conditions via effects of growth, reproduction, and survival (Westoby, 1998; Westoby and Wright, 2006; Violle et al., 2007, 2014). Many of these traits reflect the influence of evolutionary history, environmental conditions, and trade-offs in the allocation of limited resources to each component of organismal fitness. In particular, differences in plant species strategy of acquisition, use and allocation of nutrient resources have been characterized based on measures of a suite of correlated functional traits (Craine et al., 2002; Wright et al., 2004; Reich, 2014). Along a “fast-slow” plant economics spectrum, fast-growing plants are generally associated with relatively low tissue C:P and N:P ratio (i.e., higher P) and increased allocation to P rich ribosomal RNA (Elser et al., 2010). Leaves of fast growing plants also tend to be short lived and structurally flimsy, with thin lamina, low leaf mass per area (LMA), and high photosynthetic capacity and dark respiration rates (Wright et al., 2004; Elser et al., 2010; Reich, 2014). Inter-specific differences in the average plant phenotype along the “fast-slow” economic spectrum have been shown to influence the ability of different plant species to act as efficient reservoirs of plant viruses (Cronin et al., 2010), and intra-specific variation in plant functional traits in response to environmental nutrient supply could also alter epidemiological parameters (Whitaker et al., 2015).

Microbial infection also can alter host phenotype, raising the possibility for feedbacks between nutrient supply and pathogen infection on plant traits. Obligate parasites such as viruses can be considered as consumers (Aalto et al., 2015) that compete with their host for nutrient resources, which can lead to increased virulence when host resources are depleted (Smith and Holt, 1996; Smith, 2007). In this case, pathogen virulence may evolve through a trade-off, if the benefits of increased within-host replication and correlated increases in between-host transmission come at the cost of increased detrimental effects on host fitness through exploitation of host resources (Alizon et al., 2009, 2013; Froissart et al., 2010; Doumayrou et al., 2013). Ultimately, the epidemiology of horizontally transmitted pathogens could be altered by these virulence effects on host growth and lifespan, which can be approximated by several traits associated with the plant trait economics spectrum (e.g., LMA, growth rate, leaf lifespan), and this feedback may alter the interaction between plant nutrient supply rates and the growth and spread of plant pathogens.

Overall, ecological stoichiometry (Sterner and Elser, 2002) and resource competition (Miller et al., 2005) theory predict that host nutrient supplies may drive the ecology of pathogens such as plant viruses at various stages of their cycle through direct effects of the rates or ratios of available nutrients in hosts. Each stage of the epidemiological cycle also could be influenced by changes in the dynamics of coinfecting pathogens and in host functional traits mediated by host nutrient supplies. Here, we experimentally tested the effect of N and P supply rate, plant nutrient content and functional traits, and the presence of a co-infecting microbe on within-host accumulation, virulence, and between-host transmission of two plant virus species, barley yellow dwarf virus-PAV (BYDV-PAV) and cereal yellow dwarf virus-RPV (CYDV-RPV). We used a factorial combination of two nutrient supply rates of N and P that created four nutrient treatments with stoichiometric ratios replicated at low and high nutrient supply rates. By measuring plant carbon and nutrient content as well as C:N:P stoichiometry, we were able to test whether processes were primarily dependent on plant tissue content or ratios of nutrients. We tested the role of a within-host competitor on infection dynamics by including singly- and co- infected hosts. Our focal host species was *Avena sativa* (*Poaceae*), a widely-cultivated host of this virus group. We used this design to answer the following questions:

- Do plant nutrient supplies and infection alter plant stoichiometry and traits?
- Can host nutrient supplies and host tissue stoichiometry predict within-host virus titer?
- What is the relative importance of plant nutrient supplies, plant stoichiometry and traits, and coinfection on within-host virus accumulation and between-host transmission?

## Materials and methods

### Study system

Barley and cereal yellow dwarf viruses (B/CYDVs, Luteoviridae) are host generalists and are known to infect at least 150 grass species in the Poaceae family (Irwin and Thresh, 1990; D'arcy and Burnett, 1995). Infection is systemic in plants but restricted to host phloem cells. Plant infection with B/CYDVs can be associated with the expression of various symptoms, including dwarfing, yellowing and reddening, and with severe crop yield losses (Irwin and Thresh, 1990; Perry et al., 2000). B/CYDVs can also alter various plant traits such as host fecundity and longevity and have been recognized as the precursors of a dramatic shift in plant species composition in natural California grasslands (Malmstrom et al., 2005; Borer et al., 2007).

The B/CYDV group is globally distributed and includes members of the genera *Luteovirus* (e.g., BYDV-PAV) and *Polerovirus* (e.g., CYDV-RPV), two of the common B/CYDVs virus species found in both crop and wild plants (Leclercq-Le Quillec et al., 2000; Robertson and French, 2007; Seabloom et al., 2010). These viruses are obligately transmitted from plant to plant via aphid vectors (Aphididae) in a persistent, circulative, and non-propagative manner (Miller and Rasochova, 1997; Gray and Gildow, 2003). At least 25 aphid species are known as vectors of B/CYDVs, and the transmission efficiency of each virus species differs strongly among vectors (Halbert and Voegtlin, 1995; Power and Gray, 1995; Miller and Rasochova, 1997). The aphid species *Rhopalosiphum padi* is an efficient vector for both BYDV-PAV and CYDV-RPV, the focal viruses of this study.

### B/CYDVs isolates and aphid vectors

We used one isolate of each of two virus species, BYDV-PAV and CYDV-RPV that were originally collected from cereal crops in New York State and maintained in Dr. Stewart Gray's lab (Cornell University, USA). In our laboratory, we maintained these isolates by inoculating new cultures of healthy 10 day old *A. sativa* cv. Coast Black oat (Poaceae; National plant germplasm system, USDA; USA; hereafter *A. sativa*) hosts planted in 15 × 15 cm pots containing Sunshine MVP potting soil (Sun Gro Horticulture, Massachusetts, USA) every 3 weeks following the inoculation procedure described below.

Non viruliferous *R. padi* aphids were raised in 15 × 15 cm pots, each planted with 15 healthy *A. sativa* in Sunshine MVP potting soil (Sun Gro Horticulture, Massachusetts, USA). Colonies were maintained in a separate growth chamber (27°C, 16 h day, 8 h night, 32 W fluorescent bulbs) and were watered twice a week with 300 ml tap water. Approximately 100 aphids were transferred every 2 weeks to healthy 10 days old *A. sativa* plants.

### Host plant growth

Seeds of *A. sativa* were sown into 3.8 cm diameter by 21 cm depth, 164 ml pots containing a water saturated mixture of 70/30% (V/V) Sunshine, premium grade, medium vermiculite (Sun Gro Horticulture, Massachusetts, USA) and Turface MVP potting material (Turface Athletics, Illinois, USA). The pots were then placed under controlled conditions in a virus- and aphid-free growth chamber (23°C, 15 h day, 9 h night, 400 W high pressure sodium bulbs). The seeds were allowed a 10-day germination period during which seedlings were thinned to one plant per pot.

### Experimental design

Plants were randomly assigned to four groups that were mock-, singly-, or co- inoculated with BYDV-PAV or CYDV-RPV. In each group, we inoculated seven plants per fertilization treatment differing in N and P supply rate (Ctrl [7.5 μM, 1 μM]; N [375 μM, 1 μM]; P [7.5 μM, 50 μM] and NP [375 μM, 50 μM]; respectively; Table S1). The whole procedure was repeated three times. Thus, in each of our 16 experimental conditions, (4 inoculations types [Mock, BYDV-PAV, CYDV- RPV, BYDV-PAV + CYDV-RPV]) ^*^ (4 nutrient treatments [Ctrl, N, P, NP]), and due to loss of a few plants, we had between 19 and 21 plants.

Fertilization treatments represented thus a full factorial combination of two levels of N and P addition at concentrations equivalent to 0.2 and 10% of a half-strength Hoagland's nutrient solution (Hoagland and Arnon, 1938; Downs and Hellmers, 1975), a range of nutrient supply rates known to alter virus infection success in this system (Lacroix et al., 2014), while concentrations of other macro- and micro- nutrients remained constant (Table S1). Each plant was fertilized twice each week with 30 ml nutrient solution.

### Mock and virus inoculations

We performed inoculations of plants when they had two leaves following a previously published protocol (Lacroix et al., 2014) as modified from (Gray, 2008). Briefly, after a 2 h starvation period, non-viruliferous aphids were allowed a 48 h virus acquisition access period on leaves that were detached either from non-infected plants or hosts singly infected with BYDV-PAV or CYDV-RPV and that were placed in vertical 12 × 1.5 cm glass vials within a growth chamber (23°C, 16 h day, 8 h night, 40 W fluorescent bulbs). After this acquisition period, aphids that fed on detached leaves of the same infection type (non-, BYDV-PAV, and CYDV-RPV infected) were pooled together. After another 2 h starvation period, we transferred five aphids on each mock- and singly- inoculated plant and ten aphids on each co-inoculated plant (five individuals from each of the batch of aphids that fed on BYDV-PAV and CYDV-RPV infected tissue material). Aphids were enclosed into an 8 × 2.5 cm 118 μm polyester mesh cage (Sefar America Incorporated, Kansas City, Missouri, USA) affixed to the youngest leaf possible of each experimental plant (10 days old). Plants were then placed in a growth chamber (23°C, 16 h day, 8 h night, 40 W fluorescent bulbs) and aphids were killed using an insecticidal soap (Ortho) after a 72 h virus inoculation access period.

### Virus detection by RT-PCR

At 19 days post inoculation (dpi), i.e., a time point when virus infection has become systemic and virus titer is above detection threshold (Chain et al., 2005), a 20 cm piece of the first leaf of each plant was harvested and stored at −20°C for further virus detection. The infection status of each test plant was verified as described by Lacroix et al. (2014). Briefly, total RNA extraction was performed using Trizol (Invitrogen, Grand Island, NY) according to the manufacturer's instructions. RNA extracts were then stored at −20°C until use. To assess each plant infection status, we used a multiplexed RT-PCR assay that yields different fragments size for BYDV-PAV (i.e., 298 bp) and CYDV-RPV (i.e., 447 bp) using specific primers for BYDV-PAV (PAVR1, ATTGTGAAGGAATTAATGTA; PAVL1, AGAGGAGGGGCAAATCCTGT) and CYDV-RPV (RPVR2 CTGCGTTCTGACAGCAGG, RPV L ATGTTGTACCGCTTGATCCAC). These primers were adapted from a previously published protocol (Deb and Anderson, 2008). The PCR products were visualized on SybrSafe (Invitrogen) stained 2% (W/V) agarose-1000 (Invitrogen) gel using a UV-light EZ doc system (Bio-Rad) and fragment size was checked comparatively to a 100 bp DNA ladder (Apex Bioresearch Products).

### Virus titer

Within-host accumulation of each virus species was determined based on the same RNA extracts obtained as described just above and using a real-time quantification PCR protocol. Absolute quantification of virus titer was performed based on a standard curve constructed for each virus species from 10-fold serial dilutions of RNA transcripts of known concentration. These transcripts were produced using the MEGAscript® T7 Kit (Life Technologies) and amplicons obtained from regular RT-PCR with specific primers (as described above). The transcripts were then purified using a phenol/chloroform and isopropanol precipitation protocol as specified by the manufacturer's instructions. The size of the obtained transcripts was checked on a regular 2% agarose gel relatively to a 100 bp DNA ladder (Apex Bioresearch Products). RNA transcripts concentration (ng/μl) was determined using a nanodrop spectrophotometer (Thermo scientific). The obtained value was converted to mol/μl using the molecular weight of a ribonucleotide (340 g/mol) and the number of bases of each type of transcript (Nb). The following mathematical formula was applied: RNA concentration in mol/μl = RNA concentration in ng/μl ^*^ (10^−9^ ng/1 g) ^*^ (1 mol/340 g) ^*^ (1/Nb). The Avogadro's constant (6.023 × 10^23^ molecules/mol) was used to estimate the number of RNA transcript copies per μl.

TaqMan® RT-PCR reactions were performed in a final volume of 25 μl using one step RT-PCR Master Mix reagent kits (RNA-to-Ct 1-Step Kit, Applied Biosystems) and an Applied Biosystems One Plus Real-time PCR System. Three replicates of each serial dilution of RNA transcripts, of non-template control (i.e., sterile water instead of RNA), of non-amplification control (i.e., no enzymes in the reaction), and of RNA extract of each test sample were included in each run. The reactions were performed with 2.5 μl of sample (i.e., RNA extracts or sterile water) following the manufacturer's instructions. The reverse-transcription was performed during a 30 min 48°C cycle. After a 15 min 95°C activation period of the Taq polymerase, cDNA fragments were amplified following 45 cycles of denaturation (25 s 95°C), annealing and extension (1 min 60°C). cDNA amplification was performed using specific primers and probes attached to a minor groove binding (MGB) quencher and to a different reporter fluorescent dye for BYDV-PAV (forward primer, TGGTCGCCCAAAAATCTAAAAC; reverse primer GGAGTAAGGCTCGCAGTAAATTGCCGCATAAACAC; and probe, AGCAGCCTTCGTTTATCCAGTGCCAGA, FAM) and CYDV-RPV (forward primer, GAGGTTAGCGAGGAGTTAGAATTC; reverse primer, AACTACCTCAGAGTTGCCACATTC; and probe, ACATCTTCAAGACTCCTAACCTCGCCAT, VIC).

Standard curves were constructed using the known concentration of RNA transcripts (log_10_ of genomic copies in 2.5 μl) for each serial dilution and the corresponding cycle threshold (i.e., Ct, the number of amplification cycles required for a significant increase in the reporter's fluorescence). Quantification of RNA (log_10_ of genomic copies in 2.5 μl) for test samples was calculated based on the Ct threshold obtained for each sample and the standard curves. Virus titer was then expressed as the log_10_ of genomic copies per mg of fresh tissue.

### Plant traits

Plants were assessed for several functional traits that are commonly measured to describe plant species resource acquisition and use strategy along the plant economics spectrum (Craine et al., 2002; Wright et al., 2004; Reich, 2014). Measured plant traits include the number of days from mock- or virus- inoculation to the emergence of the third leaf (cf. NbDaysEmerg), the chlorophyll content of the second leaf averaged across three values per leaf measured 15 dpi with a SPAD-502 (Konica Minolta) instrument, the percent leaf area that was senescent (i.e., dry) averaged across the first and second leaf (i.e., Senes) 18 dpi, above (AG) and below-ground (BG) fresh biomass (g) and the ratio between these two values (ABG) 41 dpi, leaf dry mass per area (LMA, mg cm^−2^) and water content measured as the difference between fresh and dry leaf mass per area (mg cm^−2^) 41 dpi. We also recorded the average percent leaf area across two leaves that was covered by chlorotic symptoms characteristic of B/CYDVs infection (i.e., yellowing and reddening) 19 dpi.

Tissue chemistry data, i.e., phosphorus (P), nitrogen (N), and carbon (C) content, were obtained based on above-ground leaf tissue collected 41 dpi. For each sample, leaf tissue was oven dried at 65°C for 48 h and then ground using a Beadbeater (Biospec). For N and C content, 3 mg of dry and ground tissue from each sample was weighed in a tin capsule. N and C absolute quantification was performed using an elemental (C:H:N) analyzer. For P tissue content analysis, two replicates of 1–1.5 mg each were prepared in tin capsules. P quantification was performed using a sulfuric acid digestion protocol. A standard curve was constructed from data obtained from samples of apple NIST standard of various weights (from 0 to 6.9 mg) and known phosphorus content (i.e., 0.159% of dry tissue weight). All samples in tin capsules were introduced in previously acid washed and weighed glass vials, and were then placed in a muffle furnace for 30 min at 300°C, and then for 2 h at 550°C. Afterwards, 0.4 ml of sulfuric acid (10 N H_2_SO_4_, 10.8 M) and 5 ml of nanopure water was added to each vial. The capped vials were then autoclaved for 30 min at 121°C. Then, 1 ml of room temperature molybdate reagent (i.e., from a solution made with 0.208 g Antimony Potassium—Tartrate mixed with 9.6 g Ammonium Heptamolybdate 4-hydrate in 1 L nanopure water) was added to room temperature glass vials. Then, 0.4 ml ascorbic acid (2 g/ml) and 3.2 ml of Nanopure water was added to each vial to bring the final volume to 10 ml. Each tube was weighed and each sample was then analyzed at 880 nm using 1 cm cuvettes and a Varian spectrophotometer. N, P, and C tissue content was expressed in moles, from which tissue nutrient ratios (N:P, C:N, C:P) were calculated.

### Transmission rate

Fresh leaf tissue collected from experimental plants (i.e., source plants) 19 dpi was used to assess transmission rate. Following the inoculation protocol described above, non-viruliferous aphids were allowed a 48 h acquisition access period on leaf tissue of each source plant separately. After a 2 h starvation period, 5 aphids were transferred in a mesh cage affixed to the youngest possible leaf of each of seven 10 days old *A. sativa* recipient plants per source plant. Plants were placed in a growth chamber (23°C, 16 h day, 8 h night, 40 W fluorescent bulbs) and aphids were killed using an insecticidal soap (Ortho) after a 72 h virus inoculation access period. Recipient plants were fertilized twice a week with 30 ml of a Ctrl (7.5 μM N, 1 μM P) nutrient solution. Leaf tissue was collected 19 dpi to assess each recipient plant infection status using a RT-PCR protocol as described above. For each virus species, transmission rate was expressed as the proportion of infected recipient plants per source plant.

### Statistical analyses

All statistical analyses were performed using R version 3.3.2 (R Foundation for Statistical Computing, Vienna, Austria).

Trait data (i.e., raw values of all traits except for virus induced symptoms) of all experimental plants were analyzed using a principal component analysis (PCA) from the FactoMineR package. All plant trait variables were scaled to unit variance prior to analysis. Correlations between plant trait variables and PCA dimensions were determined based on the contribution of each variable to each dimension and based on the results (i.e., correlation coefficient and significance test) given by the dimdesc function. The principal components (i.e., orthogonal dimensions, *N* = 5) that altogether explained 80% of the variance included in the initial data set were retained for further analysis.

To assess the effect of fertilization and infection on plant traits, we analyzed the effects of host plant N and P supply rate and single- vs. co-infection on coordinates of individual plants on each of the five retained PCA dimension using model averaging (Grueber et al., 2011). This approach allowed us to take into account that explanatory variables could be covarying and that there could more than one pertinent model. All the variables were standardized prior to analysis using the standardize function in the arm R package. We used the dredge function in the MuMIn R package to fit all possible models. We estimated parameter values, errors, and AIC-weighted importance using the model.avg function in the MuMIn R package. The “Relative Importance” of each explanatory variable was estimated based on the relativized sum of the Akaike weights summed across all of the models in which the parameter appears that are within four AIC_C_ (i.e., AIC corrected for small sample size) units of the model with the lowest AIC. Importance ranges from 0 (parameter not given explanatory weight) to 1 (parameter in all top models).

For each virus species, differences in within-host accumulation in infected plants was assessed as a function of host plant N and P supply rate, the within-host presence vs. absence of a second virus species (i.e., coinfection), and plant traits as represented by coordinates of individual plants on each of the five retained PCA dimensions. We repeated this analysis of within-host titer as a function of host plant nutrient supply rates, coinfection and raw values of each measured plant trait instead of PCA coordinates. The variable corresponding to water content was removed from this latter analysis because of inherent correlation with LMA. We used a model averaging approach as described above for these two types of analyzes. We also fitted a linear model to assess the amount of virus induced symptoms, which was log_10_ transformed to normalize its distribution, as a function of within-host virus accumulation.

Finally, we assessed differences in transmission rate of each virus species separately as a function of host plant nutrient supply rate, coinfection and plant traits (i.e., represented either by coordinates of individual plants on each of the five retained PCA dimension, or raw measures of each plant trait except water) using model averaging.

## Results

### Plant traits

**Figure 1:**
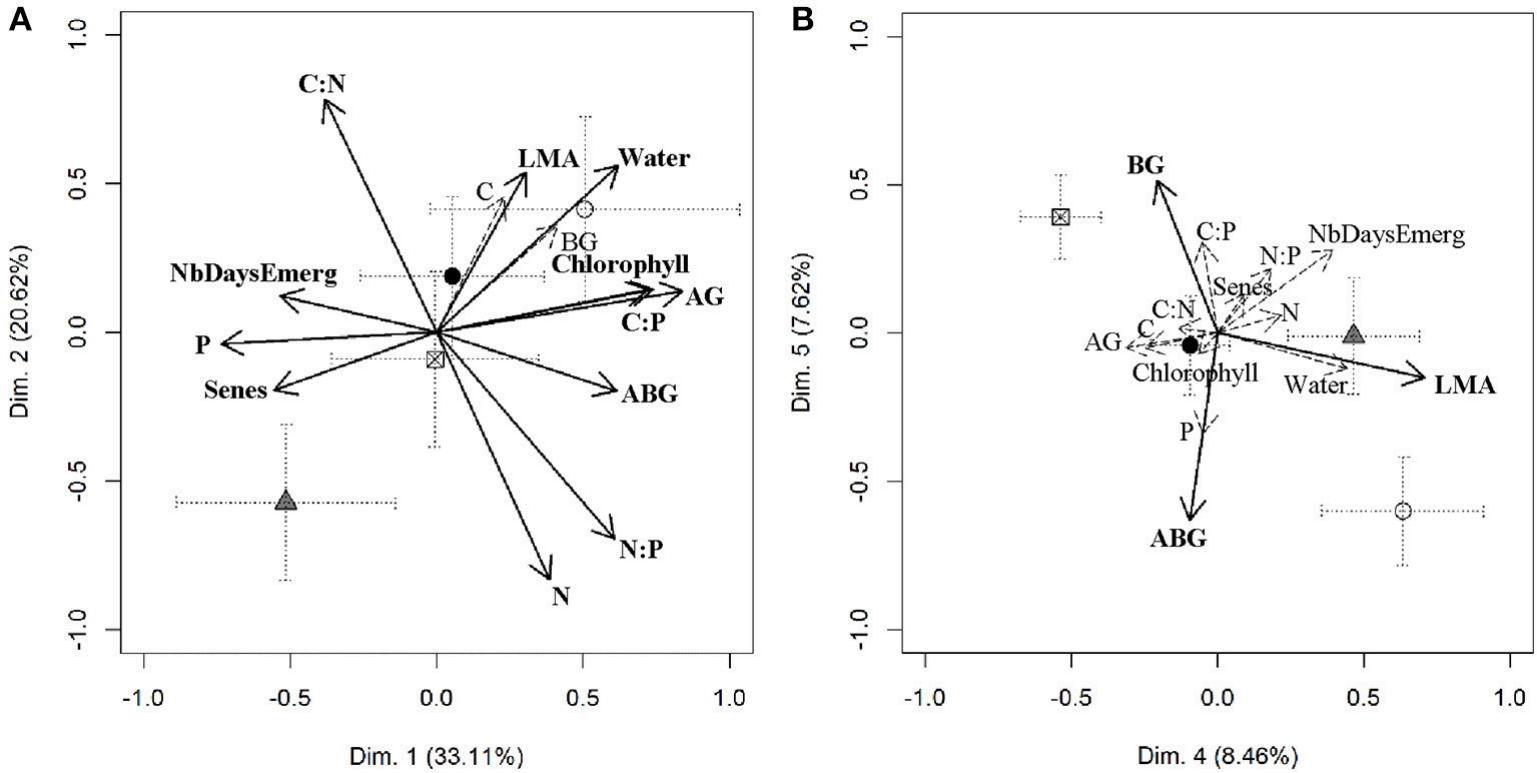
Coordinates of initial variables (i.e., plant traits illustrated with arrows) and average coordinate of non- (crossed squares), CYDV-RPV singly- (white circles), BYDV-PAV singly- (black circles), and co- (gray triangles) infected plants along principal components (Dim.) 1 and 2 **(A)** and 4 and 5 **(B)**. Measured plant traits are abbreviated as in Table 1. The vector of variables that contributed the most to Dim. 1, 2, 4, and 5 are highlighted in bold. Error bars represent ± 1 SEM.

The first five principal components, taken together, explained 80.5% of phenotypic variation included in the initial data set (Table 1). Together, the first two principal components explained 53.7% of this phenotypic variation (Table 1 and Figure 1A). Principal component 1 was associated with foliar P and above-ground growth traits. Samples with higher values on principal component 1 (i.e., Dim.1) corresponded to plants with lower P content, higher foliar C:P and N:P ratio, faster leaf emergence, higher chlorophyll content, less senescent tissue, higher above ground biomass, and above/below ground biomass ratio and higher water content (Table 1 and Figure 1A).

**Table 1:** Results of principal component analysis performed with values of traits measured on non-infected plants and plants singly- and co- infected with BYDV-PAV and/or CYDV-RPV.

	**Dim.1**	**Dim.2**	**Dim.3**	**Dim.4**	**Dim.5**
Eigenvalue	4.635	2.887	1.494	1.185	1.066
% variance	33.105	20.618	10.671	8.461	7.616
Cummulative % Variance	33.105	53.723	64.394	72.855	80.472
N	0.384	−**0.831**	0.150	0.217	0.060
P	−**0.735**	−0.039	0.480	−0.049	−0.338
C	0.229	0.456	−0.465	−0.314	−0.050
C:N	−0.382	**0.785**	−0.085	−0.136	0.016
C:P	**0.738**	0.145	−**0.525**	−0.053	0.306
N:P	**0.606**	−**0.695**	−0.070	0.183	0.216
NbDaysEmerg	−**0.534**	0.122	−0.387	0.395	0.281
Chlorophyll	**0.721**	0.143	0.162	−0.065	−0.071
Senes	−**0.554**	−0.194	−0.034	0.091	0.132
AG	**0.839**	0.139	0.306	−0.250	−0.049
BG	0.413	0.362	**0.581**	−0.206	**0.515**
ABG	**0.613**	−0.197	−0.293	−0.095	−**0.632**
LMA	0.303	**0.539**	0.105	**0.709**	−0.152
Water	**0.619**	**0.561**	0.176	0.444	−0.120

*Shown are the eigenvalue of each principal component (Dim.), the corresponding individual and cumulative percentage of variance explained, as well as the coordinates of each variable (i.e., plant trait) on each principal component. Measured plant traits correspond to average tissue content in nitrogen (N), carbon (C), and phosphorus (P) expressed in moles, tissue nutrient ratios (C:N, C:P, and N:P per mole), number of days from the mock or virus inoculation to the emergence of the third leaf (NbDaysEmerg), chlorophyll content, average percent of dry leaf area (Senes), above- (AG) and below- ground (BG) biomass (g fresh tissue) and ratio (ABG), leaf dry mass per area (LMA, mg cm^−2^) and water content (mg cm^−2^). The coordinates of the variables that contributed the most to each principal component (according to contributions of variables to each dimension and correlation tests) are highlighted in bold*.

Leaf traits, including tissue chemistry and biomass production differed along PCA dimensions 2 and 3. Plant individuals with higher coordinates on PCA dimension 2 had lower tissue N content and N:P ratio, but higher tissue C:N ratio, LMA and water content (Table 1 and Figure 1A). PCA dimension 3 explained 10.7% of phenotypic variance and contrasted high leaf tissue C:P content with high below-ground biomass (Table 1). PCA dimension 4 and 5 explained together 16.1% of phenotypic variation (Table 1 and Figure 1B). Higher values on these dimensions corresponded to individual plants with higher LMA values (cf. PCA dimension 4), and higher below-ground biomass and lower above/below ground biomass ratio (cf. PCA dimension 5; Table 1 and Figure 1B). Nutrient ratios were significantly associated with the first three PC axes, but the plant trait most strongly correlated with each PCA dimension was above-ground biomass (AG), leaf N content (N), below-ground biomass (BG), LMA, and above/below ground biomass ratio (ABG), respectively (Table 1).

### Environmental nitrogen addition and infection altered plant traits

Elevated nitrogen supply rate to host plants increased (*p* < 0.001, relative importance = 1) individual plant values on PCA dimension 1 (e.g., higher AG) but decreased (*p* = 0.006, relative importance = 1) coordinates on dimension 4 (i.e., lower LMA; Tables 2, 3). Increases in P supply rate did not significantly alter values of individual plants on any PCA dimension (Tables 2, 3).

**Table 2:** Summary of effects of N and P supply rate, single infection with BYDV-PAV or CYDV-RPV, and co-infection on plant traits (i.e., coordinates of individual plants on principal components [Dim.] 1 and 2) after model averaging.

**Response**	**Variables^a^**	**Estimate^b^**	**Std.Error**	**Adjusted SE**	**z value**	**Pr(>|z|)^c^**	**Relative Importance**	**N containing models**
Dim.1	(Intercept)	−2.326e+00	2.947e-01	2.974e-01	7.823	<2e-16	–	–
	**N_supply_**	**1.020e-02**	**8.668e-04**	**8.742e-04**	**11.665**	<2e16^***^	**1.00**	**11**
	P_supply_	−1.845e-03	6.505e-03	6.559e-03	0.281	0.779	0.36	6
	N_supply_ :P_supply_	−2.309e-05	2.784e-05	2.811e-05	0.821	0.411	0.05	1
	**PAV**	**1.314e-01**	**3.546e-01**	**3.577e-01**	**0.367**	**0.713**	**1.00**	**11**
	**RPV**	**2.046e-01**	**4.375e-01**	**4.412e-01**	**0.464**	**0.643**	**1.00**	**11**
	**PAV:RPV**	−**1.621e**+**00**	**6.233e-01**	**6.275e-01**	**2.584**	0.010^**^	**1.00**	**11**
	PAV:N_supply_	−8.329e-04	1.490e-03	1.502e-03	0.555	0.579	0.31	4
	RPV:N_supply_	1.056e-03	1.867e-03	1.881e-03	0.562	0.574	0.25	4
	PAV:P_supply_	−6.894e-03	9.496e-03	9.589e-03	0.719	0.472	0.05	1
	RPV:P_supply_	2.265e-03	1.009e-02	1.019e-02	0.222	0.824	0.04	1
	PAV:RPV:N_supply_	−4.969e-03	2.938e-03	2.967e-03	1.675	0.094	0.07	1
	PAV:RPV:P_supply_	NA	NA	NA	NA	NA	NA	NA
Dim.2	(Intercept)	1.600e-01	3.630e-01	3.653e-01	0.438	0.662	–	–
	**N_supply_**	−**1.838e-03**	**1.219e-03**	**1.227e-03**	**1.498**	**0.134**	**0.62**	**15**
	**P_supply_**	−**5.459e-04**	**1.223e-02**	**1.229e-02**	**0.044**	**0.965**	**0.64**	**16**
	N_supply_:P_supply_	6.531e-05	3.614e-05	3.649e-05	1.790	0.074	0.31	6
	PAV	1.068e-01	4.038e-01	4.065e-01	0.263	0.793	0.39	13
	RPV	2.033e-01	5.325e-01	5.357e-01	0.379	0.704	0.41	14
	**PAV:RPV**	−**1.274e**+**00**	**6.217e-01**	**6.277e-01**	**2.029**	0.042^*^	**0.23**	**8**
	PAV:N_supply_	NA	NA	NA	NA	NA	NA	NA
	RPV:N_supply_	NA	NA	NA	NA	NA	NA	NA
	PAV:P_supply_	NA	NA	NA	NA	NA	NA	NA
	RPV:P_supply_	1.308e-02	1.367e-02	1.380e-02	0.948	0.343	0.08	4
	PAV:RPV:N_supply_	NA	NA	NA	NA	NA	NA	NA
	PAV:RPV:P_supply_	NA	NA	NA	NA	NA	NA	NA

a *All variables were standardized prior to analysis. Variables of highest relative importance are highlighted in bold*.

b *NA is indicated for variables that were not included in any of the models selected within four AICc units of the model with the lowest AIC*.

c Significance of effects is indicated according to a 0.05

(*) , 0.01

(**) , and 0.001

(***) *threshold*.

**Table 3:** Summary of effects of N and P supply rate, single infection with BYDV-PAV or CYDV-RPV, and co-infection on plant traits (i.e., coordinates of individual plants on principal component [Dim.] 3, 4, and 5) after model averaging.

**Response**	**Variables^a^**	**Estimate^b^**	**Std.Error**	**Adjusted SE**	**z value**	**Pr(>|z|)^c^**	**Relative importance**	**N containing models**
Dim.3	(Intercept)	6.273e-02	2.256e-01	2.272e-01	0.276	0.782	–	–
	N_supply_	−3.109e-04	8.128e-04	8.186e-04	0.380	0.704	0.37	14
	P_supply_	6.514e-03	4.852e-03	4.896e-03	1.330	0.183	0.58	16
	N_supply_:P_supply_	−5.979e-06	2.603e-05	2.628e-05	0.228	0.820	0.02	1
	PAV	3.084e-02	3.279e-01	3.300e-01	0.093	0.926	0.38	13
	**RPV**	−**3.680e-01**	**2.916e-01**	**2.938e-01**	**1.252**	**0.210**	**0.76**	**19**
	PAV:RPV	−6.375e-01	4.443e-01	4.485e-01	1.421	0.155	0.11	3
	PAV:N_supply_	−1.925e-03	1.224e-03	1.236e-03	1.557	0.119	0.07	3
	RPV:N_supply_	3.062e-04	1.376e-03	1.389e-03	0.220	0.826	0.04	2
	PAV:P_supply_	5.609e-03	8.771e-03	8.856e-03	0.633	0.527	0.02	1
	RPV:P_supply_	2.351e-03	9.387e-03	9.477e-03	0.248	0.804	0.06	2
	PAV:RPV:N_supply_	NA	NA	NA	NA	NA	NA	NA
	PAV:RPV:P_supply_	NA	NA	NA	NA	NA	NA	NA
Dim.4	(Intercept)	−1.276e-01	2.526e-01	2.542e-01	0.502	0.616	–	–
	**N_supply_**	−**1.754e-03**	**6.324e-04**	**6.378e-04**	**2.750**	0.006^**^	**1**	**29**
	P_supply_	6.604e-03	5.943e-03	5.985e-03	1.103	0.270	0.52	19
	N_supply_:P_supply_	−3.363e-06	2.118e-05	2.138e-05	0.157	0.875	0.08	5
	**PAV**	**4.664e-01**	**2.914e-01**	**2.933e-01**	**1.590**	**0.112**	**0.80**	**24**
	**RPV**	**9.861e-01**	**3.403e-01**	**3.424e-01**	**2.880**	0.004^**^	**1**	**29**
	PAV:RPV	−5.500e-01	3.658e-01	3.693e-01	1.489	0.136	0.41	12
	PAV:N_supply_	−6.858e-04	1.014e-03	1.024e-03	0.670	0.503	0.19	8
	RPV:N_supply_	1.125e-03	1.114e-03	1.124e-03	1.001	0.317	0.3	10
	PAV:P_supply_	−1.106e-02	7.203e-03	7.273e-03	1.521	0.128	0.23	8
	RPV:P_supply_	NA	NA	NA	NA	NA	NA	NA
	PAV:RPV:N_supply_	NA	NA	NA	NA	NA	NA	NA
	PAV:RPV:P_supply_	NA	NA	NA	NA	NA	NA	NA
Dim.5	(Intercept)	6.939e-01	2.443e-01	2.462e-01	2.818	0.005	–	–
	**N_supply_**	−**9.388e-04**	**6.883e-04**	**6.940e-04**	**1.353**	**0.176**	**0.85**	**21**
	**P_supply_**	−**6.699e-03**	**5.332e-03**	**5.375e-03**	**1.246**	**0.213**	**0.71**	**19**
	N_supply_:P_supply_	−7.176e-06	2.168e-05	2.189e-05	0.328	0.743	0.10	4
	**PAV**	−**4.949e-01**	**2.731e-01**	**2.754e-01**	**1.797**	**0.072**	**1.00**	**25**
	**RPV**	−**9.257e-01**	**3.597e-01**	**3.624e-01**	**2.555**	0.011^*^	**1.00**	**25**
	**PAV:RPV**	**1.072e**+**00**	**4.171e-01**	**4.206e-01**	**2.550**	0.011^*^	**1.00**	**25**
	PAV:N_supply_	6.168e-04	1.091e-03	1.100e-03	0.561	0.575	0.25	9
	RPV:N_supply_	−1.304e-03	1.274e-03	1.285e-03	1.015	0.310	0.29	9
	PAV:P_supply_	7.094e-03	7.257e-03	7.328e-03	0.968	0.333	0.20	6
	RPV:P_supply_	1.473e-03	7.857e-03	7.932e-03	0.186	0.853	0.12	5
	PAV:RPV:N_supply_	2.935e-03	2.249e-03	2.271e-03	1.293	0.196	0.04	2
	PAV:RPV:P_supply_	NA	NA	NA	NA	NA	NA	NA

a *All variables were standardized prior to analysis. Variables of highest relative importance are highlighted in bold*.

b *NA is indicated for variables that were not included in any of the models selected within four AIC c units of the model with the lowest AIC*.

c Significance of effects is indicated according to a 0.05

(*) , 0.01

(**) , and 0.001

(***) threshold.

Single infection, either with BYDV-PAV or CYDV-RPV, did not significantly alter individual plant values on PCA dimension 1, 2, or 3 relative to mock-inoculated plants (Tables 2, 3, and Figure 1A). However, coinfection reduced plant growth and altered plant chemistry, i.e., coinfection decreased coordinates on PCA dimension 1 (*p* = 0.01, relative importance = 1, e.g., lower AG and C:P) and 2 (*p* = 0.042, relative importance = 0.23, e.g., higher N) relative to mock-inoculated plants (Table 2 and Figure 1A).

Infection by CYDV-RPV resulted in thicker (i.e., higher LMA) plant leaves, i.e., increased plant values on PCA dimension 4 (*p* = 0.004, relative importance = 0.1), and coinfection did not alter this relationship (Table 3 and Figure 1B). In addition, CYDV-RPV infected plants were characterized by a higher above- to below- ground biomass ratio, i.e., associated with significantly lower coordinates on PCA dimension 5 (*p* = 0.011, relative importance = 1). However, the presence of BYDV-PAV increased coordinates of CYDV-RPV infected hosts (*p* = 0.011, relative importance = 1; Table 3 and Figure 1B) on PCA dimension 5. Traits of BYDV-PAV infected plants did not differ from mock-inoculated plants on PCA dimensions 4 and 5 (Table 3 and Figure 1B).

### CYDV-RPV, but not BYDV-PAV, within-host accumulation was correlated with changes in plant traits

**Figure 2:**
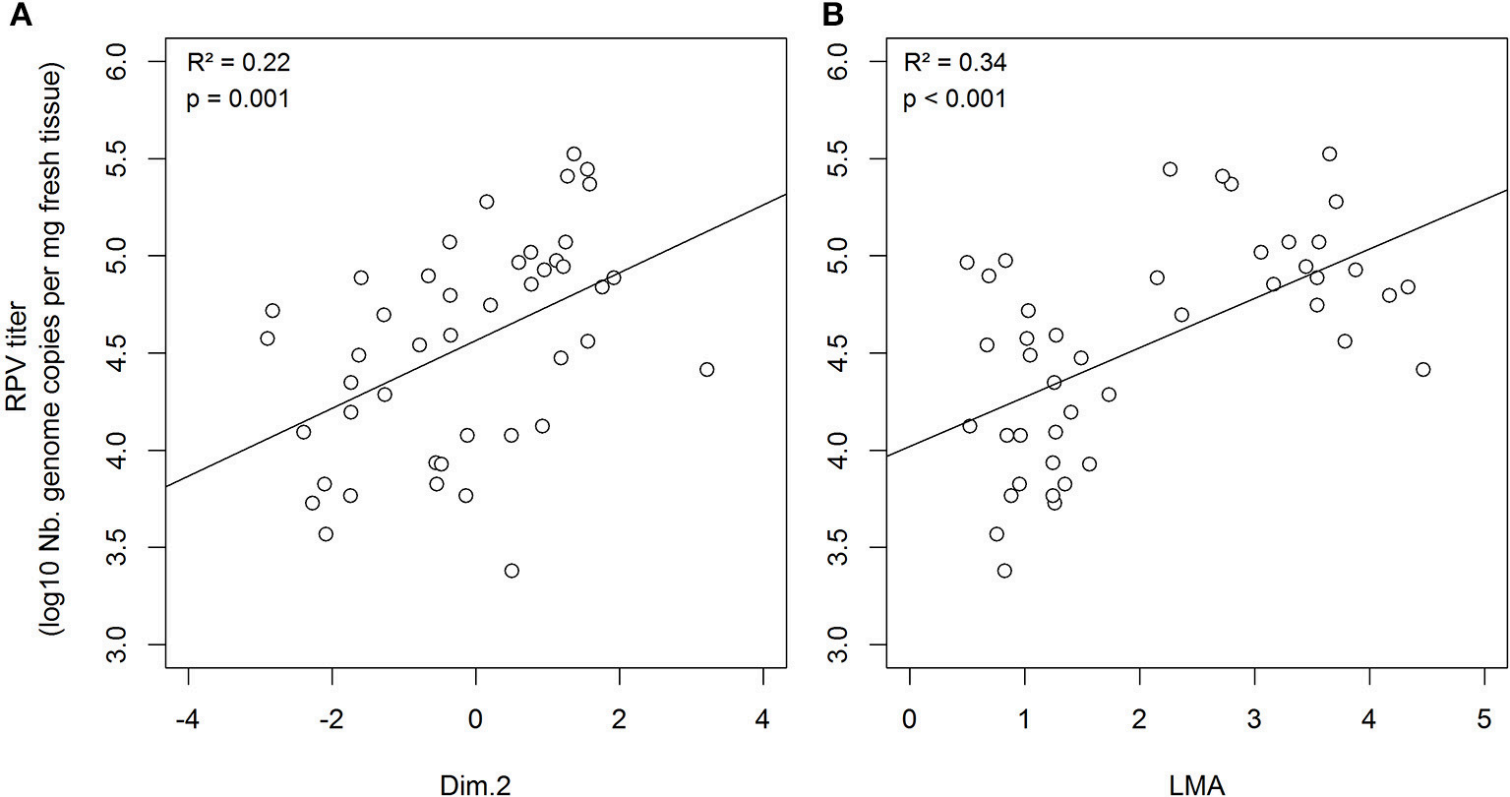
Within-host CYDV-RPV accumulation measured based on leaf tissue 19 dpi as a function of individual plants coordinates on the second principal component **(A)** and of individual plant LMA values **(B)**. The R-square, *p*-value and slope is shown for significant linear bivariate relationships.

**Figure 3:**
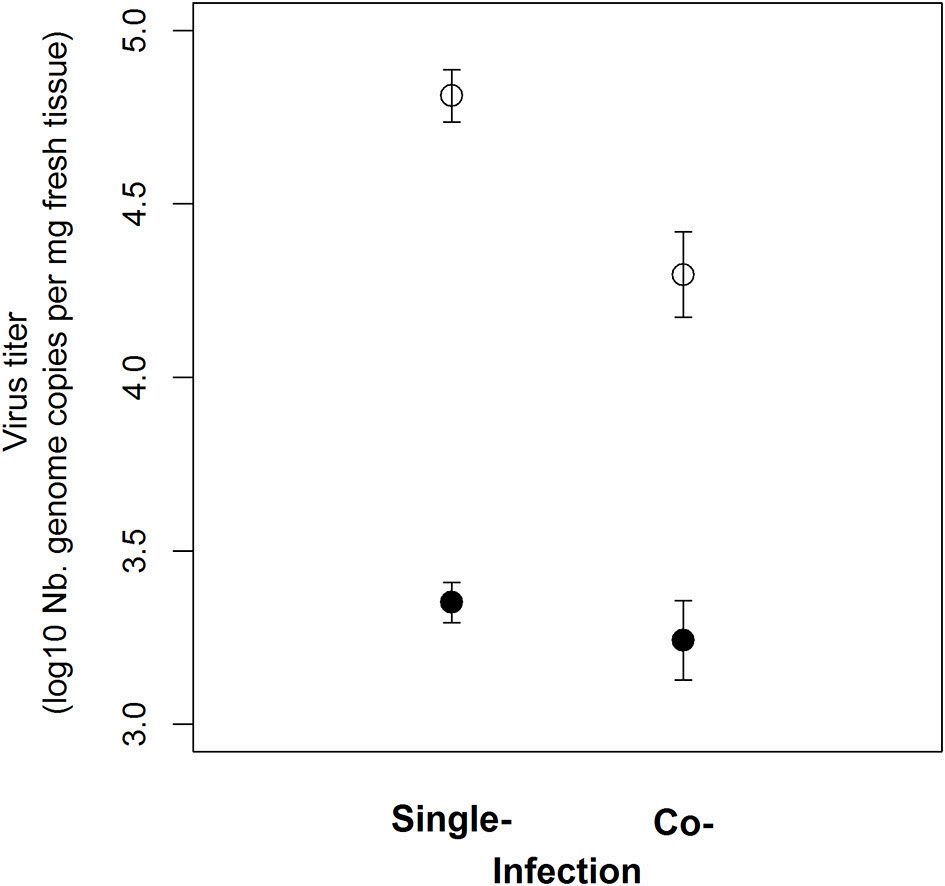
Average virus accumulation in leaf tissue 19 dpi for CYDV-RPV (white circles) and BYDV-PAV (black circles) in single- and co-infection. Error bars represent ± 1 SEM.

Elevated N and P supply rates did not directly alter the within-host accumulation of CYDV-RPV (Tables 4, 5). However, CYDV-RPV titer was increased within plants with higher coordinates PCA dimension 2 (e.g., higher plant C:N and LMA, *p* = 0.039, relative importance = 0.81, Table 4 and Figure 2A). A detailed analysis of CYDV-RPV within-host accumulation as a function of nutrient supply rate, coinfection, and values of each plant trait revealed a positive correlation with LMA (*p* = 0.001, relative importance = 1; Table 5 and Figure 2B), and a negative effect of coinfection (*p* = 0.025, relative importance = 0.91; Table 5 and Figure 3). BYDV-PAV titer was not affected by environmental supply rates, co-infection, or plant traits (Tables 4, 5 and Figure 3).

**Table 4:** Summary of effects of N and P supply rate, co-infection and plant traits (i.e., coordinates of individual plants on each principal component [Dim.]) on CYDV-RPV and BYDV-PAV titer after model averaging.

**Response**	**Variables^a^**	**Estimate**	**Std. Error**	**AdjustedSE**	**Z value**	**Pr(>|z|)^b^**	**Relative importance**	**N containing models**
RPV Titer	(Intercept)	4.54e+00	6.43e-02	6.63e-02	68.484	<2e-16	–	–
	**N_supply_**	−**1.55e-03**	**7.86e-04**	**8.01e-04**	**1.93**	**0.054**	**0.67**	**23**
	P_supply_	−1.29e-03	3.32e-03	3.42e-03	0.377	0.706	0.10	5
	Coinfection	−3.16e-01	1.70e-01	1.75e-01	1.808	0.071	0.58	20
	Dim.1	5.06e-01	2.61e-01	2.66e-01	1.903	0.057	0.59	20
	**Dim.2**	**3.24e-01**	**1.53e-01**	**1.58e-01**	**2.054**	0.039^*^	**0.81**	**25**
	Dim.3	3.24e-02	1.80e-01	1.84e-01	0.176	0.861	0.14	7
	**Dim.4**	**2.87e-01**	**1.53e-01**	**1.57e-01**	**1.823**	**0.068**	**0.65**	**23**
	Dim.5	−2.85e-01	1.82e-01	1.87e-01	1.521	0.128	0.52	20
PAV Titer	(Intercept)	3.32e+00	5.40e-02	5.53e-02	59.903	<2e-16	–	–
	N_supply_	2.35e-06	4.18e-04	4.26e-04	0.006	0.996	0.12	10
	P_supply_	−2.92e-03	2.24e-03	2.29e-03	1.274	0.203	0.39	26
	Coinfection	−1.77e-01	1.37e-01	1.40e-01	1.263	0.207	0.40	27
	Dim.1	1.11e-01	1.22e-01	1.25e-01	0.893	0.372	0.12	15
	Dim.2	8.68e-02	1.11e-01	1.14e-01	0.763	0.445	0.17	13
	Dim.3	−1.20e-01	1.36e-01	1.39e-01	0.866	0.386	0.19	13
	Dim.4	1.49e-01	1.20e-01	1.22e-01	1.215	0.224	0.36	24
	Dim.5	−1.10e-01	1.16e-01	1.19e-01	0.93	0.353	0.22	15

a *All variables were standardized prior to analysis. Variables of highest relative importance are highlighted in bold*.

b Significance of effects is indicated according to a 0.05

(*) , 0.01

(**), and 0.001 (***) threshold.

**Table 5:** Summary of effects of N and P supply rate, co-infection and individual plant traits on CYDV-RPV and BYDV-PAV titer after model averaging.

**Response**	**Variables^a^^,^^b^**	**Estimate**	**Std. Error**	**AdjustedSE**	**Z value**	**Pr(>|z|)^c^**	**Relative importance**	**N Containing models**
RPV Titer	(Intercept)	4.542	0.063	0.065	70.426	−2e–16^***^	–	–
	Nsupply	−0.001	0.001	0.001	1.291	0.197	0.27	45
	Psupply	−0.002	0.003	0.003	0.727	0.467	0.08	13
	**Coinfection**	−**0.339**	**0.147**	**0.151**	**2.238**	0.025^*^	**0.91**	**129**
	C	−0.188	0.166	0.171	1.103	0.270	0.17	28
	N	−0.637	0.555	0.561	1.136	0.256	0.52	74
	P	−0.063	0.444	0.450	0.140	0.889	0.08	15
	CN	0.202	0.197	0.202	1.003	0.316	0.22	35
	CP	−0.429	0.292	0.298	1.441	0.150	0.26	37
	NP	0.756	0.586	0.596	1.269	0.204	0.29	42
	ABG	0.196	0.223	0.228	0.858	0.391	0.09	15
	AG	0.157	0.277	0.281	0.561	0.575	0.10	19
	BG	−0.152	0.164	0.169	0.902	0.367	0.09	15
	Chlorophyll	−0.026	0.151	0.155	0.166	0.868	0.05	10
	**LMA**	**0.520**	**0.151**	**0.155**	**3.351**	0.001^***^	**1.00**	**146**
	NbDaysEmerg	0.006	0.146	0.150	0.040	0.968	0.04	7
	Senes	−0.205	0.146	0.151	1.360	0.174	0.32	49
PAV Titer	(Intercept)	3.315	0.053	0.054	60.949	<2e-16^***^	–	–
	Nsupply	−0.001	0.001	0.001	1.459	0.145	0.33	111
	Psupply	−0.003	0.002	0.002	1.281	0.200	0.30	109
	Coinfection	−0.167	0.146	0.149	1.116	0.265	0.18	67
	C	0.121	0.112	0.115	1.051	0.293	0.15	53
	N	−0.092	0.162	0.165	0.559	0.576	0.06	26
	P	0.001	0.161	0.164	0.005	0.996	0.05	22
	CN	−0.122	0.150	0.153	0.798	0.425	0.10	42
	CP	0.109	0.143	0.146	0.750	0.453	0.08	33
	NP	0.006	0.119	0.122	0.051	0.959	0.04	16
	ABG	0.065	0.222	0.225	0.290	0.772	0.17	65
	AG	0.397	0.283	0.286	1.388	0.165	0.62	215
	BG	−0.262	0.204	0.208	1.258	0.209	0.30	105
	Chlorophyll	−0.167	0.148	0.151	1.103	0.270	0.18	68
	LMA	0.159	0.113	0.115	1.378	0.168	0.38	138
	NbDaysEmerg	0.213	0.141	0.144	1.483	0.138	0.37	124
	Senes	0.003	0.135	0.18	0.021	0.983	0.04	19

a *All variables were standardized prior to analysis. Variables of highest relative importance are highlighted in bold*.

b *Because leaf water content was calculated based on fresh mass per area and LMA, the variable water content was removed from this analysis to avoid inherent correlations in explanatory variables*.

c Significance of effects is indicated according to a 0.05

(*) , 0.01

(**), and 0.001

(***) *threshold*.

### CYDV-RPV within-host accumulation was correlated with chlorotic symptoms

**Figure 4:**
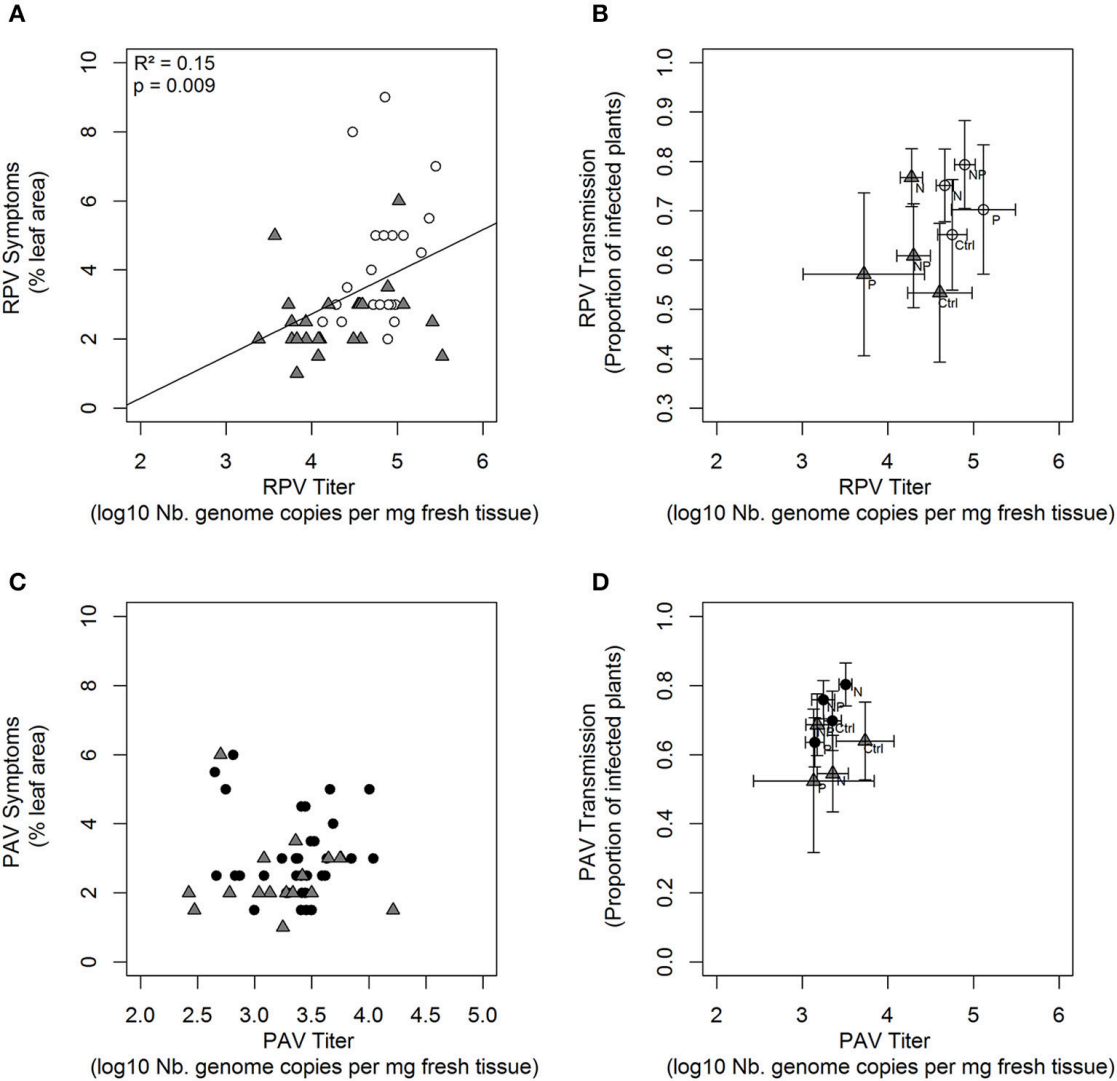
Percent leaf area displaying symptoms associated with RPV **(A)** and PAV infection **(C)** and average proportion of plants infected after secondary transmission from differentially fertilized test plants as a function of RPV **(B)** and PAV **(D)** within-host accumulation measured based on leaf tissue 19 dpi. Virus associated titer, symptoms and transmission rate is shown for “source” RPV singly- (white circles), PAV singly- (black circles), and co- (gray triangles) infected plants. The R-square, *p*-value and line is shown for significant linear bivariate relationships. Nitrogen and phosphorus content of fertilization solutions Ctrl (Control), N (Nitrogen), P (Phosphorus), and NP (Nitrogen and Phosphorus) are as follows: 7.5/1, 375/1, 7.5/50, 375/50 μM, respectively. Error bars represent ± 1 SEM.

The percent area of infected leaf that was discolored (i.e., yellowing and reddening) was positively correlated with within-host titer for CYDV-RPV (*p* = 0.004, Figure 4A) but not for BYDV-PAV (*p* = 0.95, Figure 4C). Finally, within-host titer did not directly affect CYDV-RPV and BYDV-PAV virus transmission rate (Tables S2, S3 and Figures 4B,D). In addition, virus transmission rate was not significantly affected by nutrient supply rates, tissue chemistry, virus coinfection, or plant traits (Tables S2, S3).

## Discussion

The ecology of plant viruses may be influenced at various stages of their epidemiological cycle by multiple abiotic and biotic factors. The pathways by which nutrients influence virus population growth and transmission can include both direct effects of elevated host nutrient supplies and content on virus infection success and growth and indirect effects of nutrient addition through alterations of coinfecting pathogen dynamics and host phenotype. In contrast to predictions of ecological stoichiometry (Sterner and Elser, 2002) and resource competition (Miller et al., 2005) theories, we did not find any direct effects of rates and ratios of nutrients in environmental supplies or plant tissue on virus dynamics in our system. Increased nitrogen supply shifted plant phenotype toward higher plant growth rate and thinner leaves (i.e., reduced LMA), consistently with expected effects of N fertilization (Elser et al., 2007; Dordas, 2009; Reich, 2014), while infection with CYDV-RPV resulted in thicker plant leaves (i.e., higher LMA). CYDV-RPV titer was higher in plants with higher LMA. CYDV-RPV titer also was reduced by the presence of BYDV-PAV, further indicating within-host competition between these closely-related viruses (Lacroix et al., 2014). Chlorotic symptoms increased with CYDV-RPV titer, but transmission rate was independent of nutrient supply, tissue stoichiometry, structural plant traits, and virus titer. Our results reveal a more complex relationship between environmental nutrient supply, virus dynamics, alterations of plant phenotype, and within-host competition among pathogens.

### Environmental nutrient supplies and plant tissue chemistry did not directly alter virus titer

Ecological stoichiometry theory predicts that ratios in environmental nutrient supply or host nutrient content will limit infection success and the production of N and P demanding virus particles (Sterner and Elser, 2002), whereas resource competition theory predicts population growth, persistence and species coexistence based on both nutrient rates and ratios. Nutrient supply rates and ratios, in particular in carbon, nitrogen, and phosphorus, can limit virus multiplication in algae and phytoplankton hosts (Elser et al., 2003; Clasen and Elser, 2007; Frost et al., 2008; Maat and Brussaard, 2016). In contrast to these predictions and the tests in aquatic ecosystems, we did not find any direct effects of nutrient supplies or plant tissue stoichiometry on the density (i.e., titer) of the two virus species examined in our study. Our study shows that alterations of plant phenotypic traits may be a key connection between nutrient rates and ratios and epidemiological rates.

### Infection and environmental nutrient supplies altered plant phenotype

In our study, BYDV-PAV and CYDV-RPV did not reduce above-ground biomass, which contrasts with other studies of B/CYDV infection (Catherall, 1966; Malmstrom et al., 2005; Mordecai et al., 2015). However, we did find that leaf mass per area (i.e., LMA) was higher in CYDV-RPV infected plants, that CYDV-RPV titer was higher in plants with high LMA leaves, and that chlorotic symptoms induced on leaves increased with CYDV-RPV titer. Previous work on this virus group found reductions in fresh biomass and in yield of grass hosts infected with B/CYDV, and infected hosts also were characterized by higher leaf dry weight, which is similar to our findings for CYDV-RPV infected plants (Jensen, 1968, 1969, 1972). B/CYDV effects on plant phenotype arise from reduced translocation of photosynthates from leaves to the roots and apical meristem, resulting in the accumulation of soluble carbohydrates, starch and non-soluble proteins in diseased leaves, increased dry weight and dark respiration rates, decreased photosynthesis, and induction of chlorotic symptoms (Jensen, 1968, 1969, 1972; Malmstrom and Field, 1997). The increased non-soluble protein concentration in infected leaves may be due to the accumulation of non-soluble proteins, free amino acids, nucleic acids, amines, amides, and inorganic nitrogen (Jensen, 1969). These results in combination with our study suggest that the higher LMA associated with CYDV-RPV infection observed here could result from reduced nutrient translocation and subsequent accumulation of C-rich (e.g., carbohydrates and starch) and N-rich (e.g., non-soluble protein fraction) compounds in infected leaves. The accumulation of these resources in leaves of infected plants could induce stronger changes in phenotypes than nutrient supplies and could facilitate the CYDV-RPV multiplication cycle, increase virus titer, and lead to reduced photosynthate production and translocation to other plant parts causing chlorotic symptoms.

### Possible role of carbon and nitrogen rich compounds in plant tissues on virus titer

Our work further suggests that CYDV-RPV replication may not be directly limited by total tissue N, P, and C content and ratios, but by the concentration and ratios of specific C- and/ or N-rich molecules. Although, B/CYDV infection disrupts nutrient translocation and increases the content of C- and N- rich compounds in infected leaves, a greater amount of the carbohydrates accumulated during the day in leaves of infected vs. uninfected plants grown under elevated CO_2_ conditions have been shown to be converted, exported and/or respired during the night due to decreases in sucrose, glucose and fructose concentration (Malmstrom and Field, 1997). This suggests that sugars could have been reallocated to fuel virus multiplication. Within-host BYDV-PAV accumulation was further found to increase in host plants exposed to elevated CO_2_ levels, although this increase was unrelated to either increased plant growth or to the absolute dry weight (g) or mean leaf area (cm^−2^) (Trebicki et al., 2015). However, specific leaf area (cm^2^ g^−1^) was lower (i.e., higher LMA) in infected compared to uninfected plants (Trebicki et al., 2015), suggesting a potential positive correlation between BYDV-PAV titer and LMA. In contrast, increased host growth under conditions of elevated N supply led to increased BYDV-PAV titer in a different study (Whitaker et al., 2015), although this only occurred in small plants and this effect was reversed in larger plants (Whitaker et al., 2015), perhaps due to reduced allocation of nutrients by plants to cellular machinery with increasing plant size (Elser et al., 2010). Finally, carbohydrates (e.g., fructose, mannitol, and trehalose) were higher in both healthy and B/CYDV infected plants under elevated CO_2_ levels, and amino acid concentrations were higher in infected plants irrespective of atmospheric CO_2_ levels (Vassiliadis et al., 2016). Taken together, these studies suggest that the titer of B/CYDVs may rely more on the content and ratios of particular C-rich (e.g., sugars like sucrose and fructose) and N-rich (e.g., amino acids) molecules than elemental nutrient concentration *per se*. The joint analysis of metabolic profiles in infected and healthy plants, of within-host virus accumulation and between-host transmission, and of the molecular mechanisms underlying these processes thus likely constitute a fruitful avenue for research.

### Virus and plant species differences in functional traits might drive infection and plant responses to environmental nutrient supplies

We did not find any relationship between BYDV-PAV titer or transmission rate and environmental host nutrient supplies, plant traits, or stoichiometry. In our experimental conditions, environmental and host tissue nutrient rates and ratios may not have been limiting for the BYDV-PAV multiplication cycle. As suggested previously, the question of potential differences in nutrient requirements for both CYDV-RPV and BYDV-PAV virus species remains open, and limitations in C-rich and N-rich metabolites might be stronger for CYDV-RPV than for BYDV-PAV (Lacroix et al., 2014; Smith, 2014). Moreover, Cronin et al. (2010) found inter-specific differences in plant species ability to act as reservoirs of BYDV-PAV, such that host susceptibility to the inoculation, within-host accumulation, and transmission rate of BYDV-PAV was higher on average for plant species characterized by a “faster” plant phenotype (Cronin et al., 2010). In addition, while previous field experiments have demonstrated a positive effect of P supply and N:P ratio on B/CYDV prevalence (Borer et al., 2010, 2014b), elevated P supply to grass hosts in controlled conditions decreased BYDV-PAV within-host accumulation in *Avena fatua* but not in *Bromus hordeaceus* grass hosts (Poaceae) (Rua et al., 2013). In our study, we found differences in the effects of CYDV-RPV- single, BYDV-PAV- single, and coinfection on plant phenotype, consistent with the emerging perspective of the diversity of possible host-virus interactions (Marquez et al., 2007; Roossinck et al., 2010; Roossinck, 2015). Although, BYDV-PAV alone did not significantly alter plant phenotype, coinfected plants were characterized by higher LMA, reduced above- to below- ground biomass ratio and plant growth, higher N and P content; higher N:P ratio; and more senescent tissue than uninfected or CYDV-RPV singly-infected hosts, suggesting that coinfection reduced plants' ability to allocate resources to plant growth. Overall, these results suggest that plant responses to infection, as well as virus epidemiological parameters at various stages of infection might be influenced by multiple factors including inter- and intra-specific virus and plant differences in functional traits, plant nutrient and metabolite stoichiometry, and environmental abiotic conditions.

### Environmental nutrient supplies may differentially alter various steps of the epidemiological cycle

In terrestrial systems, elevated environmental nutrient supplies to grass species in natural ecosystems have been shown to increase the prevalence and co-infection rates of a group of generalist and aphid-vectored plant viruses (i.e., barley and cereal yellow dwarf viruses; Seabloom et al., 2009, 2013; Borer et al., 2010, 2014a,b). These responses observed in natural conditions may be the result of multiple interacting processes such as changes in the host plant community (Borer et al., 2009, 2010), altered vector behavior or performance (Borer et al., 2009; Seabloom et al., 2013), direct influences of plant nutrient supplies and plant stoichiometry on host susceptibility, virus inter-specific interactions, virus multiplication, and transmission (Smith, 1993, 2007; Smith and Holt, 1996), or through indirect influences on plant species diversity, composition, and functional traits (Liu et al., 2017).

Our work and that presented by Lacroix et al. (2014) now provide deeper insight into the host-level mechanisms that underlie nutrient effects on pathogen spread observed in natural ecosystems. Importantly, we have shown that infection success was differentially altered by nutrient supply rates and virus competition (Lacroix et al., 2014). CYDV-RPV infection rates were reduced by both P supply rates and competition with BYDV-PAV, but only at low N supply rates. After the establishment of infection, this study shows CYDV-RPV titer was affected through changes in plant phenotype, possibly through changes in rates and ratios of C-rich and N-rich metabolites, and titer was reduced by the presence of a competitor (coinfection). Higher virus titer led to increased expression of chlorotic symptoms, at least for CYDV-RPV. In our conditions, transmission, the final step in the epidemiological cycle, was independent of nutrient supplies, host phenotype, virus coinfection, and virus titer. The transmission experiment in our study was designed to assess effects of environmental nutrient supplies on host-to-host transmission through alterations of virus titer. Virus titer in all hosts and/or the acquisition access period allowed for the aphids to acquire virus particles from host tissue may have been high enough to maximize between-host transmission rate in our experimental conditions, which could explain the absence of correlation between titer and transmission often observed in plant-virus systems (Froissart et al., 2010). In addition to virus titer, host-to-host virus transmission can be strongly influenced by aphid vector behavior, especially for persistently transmitted viruses that require long acquisition time by aphids (Froissart et al., 2010; Ingwell et al., 2012; Smith, 2014; Blanc and Michalakis, 2016). Further studies investigating whether environmental nutrient supplies could influence among host virus transmission through alterations of host attractiveness to insect vectors and aphid feeding behavior could deepen our knowledge on the multiple pathways through which host nutrient resources could influence plant virus dynamics.

## General conclusions

Our work highlights the challenges of understanding the implications of elevated nutrient deposition and altered global biogeochemical cycles (Tilman et al., 2001; Rockström et al., 2009) on disease ecology and epidemiology. While ecological stoichiometry and resource competition theory can provide a starting point to understand the effects of altered nutrient supply rates and ratios on disease and can effectively predict some of the links in the transmission chain, our work highlights the importance of disentangling the role of specific C-rich and N-rich metabolites on plant virus titer, rather than focusing solely on total elemental N, P, and C supply, content, or ratio. In addition, this work demonstrates that it is critical to understand the molecular mechanisms that lead to the host phenotypic changes that underlie host-virus interactions. With this stronger understanding of the direct and indirect pathways by which nutrient supply rates and ratios influence pathogen dynamics, we continue to build a predictive understanding of the effects of changing environmental conditions on virus multiplication and transmission.

## Author contributions

CL, ES, and EB conceived and designed this research project. CL performed the experiments with the help of other lab members. CL analyzed data and drafted the paper, and ES and EB provided substantial contributions for statistical analysis and manuscript revisions. All authors approved this version of the manuscript and agreed to be accountable for all aspects of this work.

### Conflict of interest statement

The authors declare that the research was conducted in the absence of any commercial or financial relationships that could be construed as a potential conflict of interest.
