# Supplementary material for: Environmental Nutrient Supply Directly Alters Plant Traits but Indirectly Determines Virus Growth Rate
Source: Front Microbiol. 2017 Nov 6;8:2116. doi: 10.3389/fmicb.2017.02116 (PMC5681519; doi:10.3389/fmicb.2017.02116)
Supplement: Supplementary file 1 [file Table1.DOCX]

**Environmental nutrient supply directly alters plant traits but indirectly determines virus growth rate**

**Christelle Lacroix^*#^, Eric W. Seabloom and Elizabeth T. Borer**

*Department of Ecology, Evolution, and Behavior, University of Minnesota, Saint Paul, MN, USA*

***Correspondence:**

Christelle Lacroix

christelle.lacroix@paca.inra.fr

**# Present address:**

***UR0407 Plant Pathology, INRA, Montfavet, France***

**SUPPLEMENTARY TABLES**

**Table S1. Characteristics of nutrients included in fertilization solutions.**

^a^ Each macro- (other than phosphorus and nitrogen) and micro- nutrient concentration remained constant across fertilization solutions.

^b^ Phosphorus (KH_2_PO_4_) and nitrogen (NH_4_NO_3_) were supplied at concentrations equivalent to 0.2% (1 and 7.5µM. respectively) and 10% (50 and 375µM. respectively) of a half-strength Hoagland’s solution (Hoagland and Arnon. 1938). These concentrations were crossed to produce four nutrient solutions.
