# Supplementary material for: Environmental Nutrient Supply Directly Alters Plant Traits but Indirectly Determines Virus Growth Rate
Source: Front Microbiol. 2017 Nov 6;8:2116. doi: 10.3389/fmicb.2017.02116 (PMC5681519; doi:10.3389/fmicb.2017.02116)
Supplement: Supplementary file 2 [file Table2.DOCX]

**Environmental nutrient supply directly alters plant traits but indirectly determines virus growth rate**

**Christelle Lacroix^*#^, Eric W. Seabloom and Elizabeth T. Borer**

*Department of Ecology, Evolution, and Behavior, University of Minnesota, Saint Paul, MN, USA*

***Correspondence:**

Christelle Lacroix

christelle.lacroix@paca.inra.fr

**# Present address:**

***UR0407 Plant Pathology, INRA, Montfavet, France***

**SUPPLEMENTARY TABLES**

**Table S2. Summary of effects of N and P supply rate, co-infection and plant traits (i.e. coordinates of individual plants on each principal component [Dim.]) on virus transmission rate after model averaging. All variables were standardized prior to analysis.**

^a^ Significance of effects is indicated according to a 0.05 (*), 0.01 (**) and 0.001 (***) threshold.
